# Antibiotic Selection Pressure and Resistance in *Streptococcus pneumoniae* and *Streptococcus pyogenes*

**DOI:** 10.3201/eid1003.030252

**Published:** 2004-03

**Authors:** Werner C. Albrich, Dominique L. Monnet, Stephan Harbarth

**Affiliations:** * Emory University School of Medicine, Atlanta, Georgia, USA; † Statens Serum Institut, Copenhagen, Denmark; ‡ University of Geneva Hospitals, Geneva, Switzerland

**Keywords:** antibiotics, drug resistance, microbial, epidemiology, *Streptococcus pneumoniae*, *Streptococcus pyogenes*

## Abstract

We correlated outpatient antibiotic use with prevalence of penicillin-nonsusceptible *Streptococcus pneumoniae* (PNSP), macrolide-resistant *S. pneumoniae* (MRSP), and macrolide-resistant *S. pyogenes* (MRGAS) in 20 countries. Total antibiotic use was correlated with PNSP (r = 0.75; p < 0.001), as was macrolide use with MRSP (r = 0.88; p < 0.001) and MRGAS (r = 0.71; p = 0.004). Streptococcal resistance is directly associated with antibiotic selection pressure on a national level.

A global trend of increasing antimicrobial resistance, but with wide variations at national levels, is well-documented in the literature (*1*). Strong evidence supports an association between antibiotic use and resistance in hospitals (*2*,*3*). By contrast, the relationship between antibiotic consumption and resistance has been more difficult to establish for the outpatient setting, although some data suggest a direct correlation for streptococcal infections (*4*–*6*). For instance, an ecologic study linked penicillin-nonsusceptibility in *Streptococcus* *pneumoniae* with β-lactam and macrolide use in 12 European countries (*7*). Recently, McCormick et al. have shown that variation in pneumococcal resistance in the United States is best explained by geographic variation in antibiotic selection pressure, rather than by clonal dynamics (*8*).

Penicillin-nonsusceptible *S*. *pneumoniae* (PNSP) and macrolide-resistant *S. pneumoniae* (MRSP) are markers of resistance to antibiotics commonly used as first-line drugs for respiratory tract infections. Although penicillin-resistant *S*. *pyogenes* has never been observed to date, the increasing rates of macrolide-resistant group A streptococci (MRGAS) pose considerable clinical problems in many countries (*4*). The aim of this ecologic study was to correlate outpatient antibiotic consumption with reported rates of PNSP, MRSP, and MRGAS in 20 countries on three continents.

## The Study

An ecologic study design was used, which allows measurement of the total (individual and group-level) effect of antibiotic exposure on antimicrobial resistance in streptococci (*9*). PNSP, MRSP, and MRGAS were chosen as indicator organisms for those effects. National outpatient antibiotic sales data were obtained from published reports, provided by IMS Health Global Services, the National Corporation of Swedish Pharmacies, the Danish Medicines Agency, and the Institute of Public Health in Slovenia (*7*,*10*–*13*). Data for the United States were extracted from IMS data (*10*). Sales data for total antibiotic use (anatomic therapeutic classification [ATC] group J01) and macrolide use (ATC group J01F) were used to express outpatient consumption in defined daily doses (DDD) per 1,000 inhabitants per day, as recommended by the World Health Organization (WHO) (*14*).

By using MEDLINE, we performed a systematic search of national and international surveillance studies published in English, French, or German that reported proportional frequencies of PNSP, MRSP, and MRGAS for 1994 to 2000. Representative resistance data from those countries were included for which antibiotic sales data were also available (*6*,*7*,*10*,*13*,*15*–*34*). A time lag of 0 to 2 years between antibiotic sales and ensuing antimicrobial resistance data was considered acceptable for this study. For three countries (Iceland, Finland, and Sweden), the lag time between antibiotic sales data and resistance rates was 0. Studies limited to small geographic areas not representative of a given country and studies limited to particular patient populations were excluded. Nonsusceptibility to penicillin and macrolide resistance included intermediate and high-level resistance.

The relationship between total outpatient antibiotic consumption and rates of PNSP was analyzed for 20 countries (Australia, Austria, Belgium, Canada, Denmark, Finland, France, Germany, Greece, Iceland, Ireland, Italy, Luxembourg, Netherlands, Norway, Portugal, Spain, Sweden, United Kingdom, and United States). We also calculated relationships between macrolide use and MRSP for 16 countries (Australia, Austria, Belgium, Denmark, Finland, France, Germany, Greece, Ireland, Italy, Netherlands, Portugal, Slovenia, Spain, Sweden, and United Kingdom), and between macrolide use and MRGAS for 14 countries (Australia, Austria, Belgium, Finland, France, Germany, Greece, Italy, Netherlands, Portugal, Slovenia, Spain, Sweden, and United Kingdom). For these correlation analyses, we used two-tailed Spearman coefficients (r) for nonparametric correlations.

**Figure 1:**
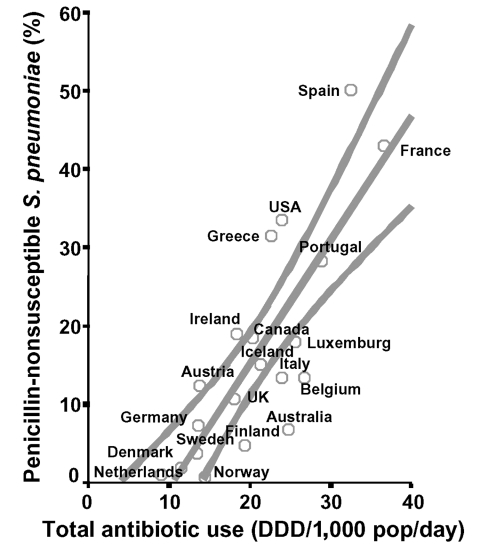
Total antibiotic use in the outpatient setting (vertical axis) versus prevalence of penicillin-nonsusceptible *Streptococcus pneumoniae* (horizontal axis) in 20 industrialized countries. A regression line was fitted with 95% confidence bands (r = 0.75; p < 0.001).

**Figure 2:**
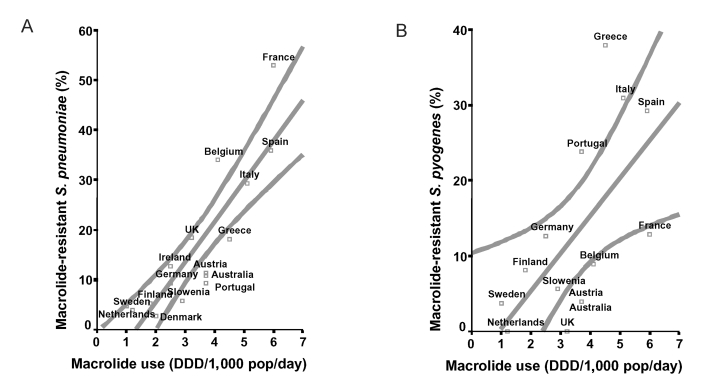
A. Relationship between macrolide use in the outpatient setting (horizontal axis) and prevalence of macrolide-resistant *Streptococcus pneumoniae* (vertical axis) in 16 industrialized countries. A regression line was fitted with 95% confidence bands (r = 0.88; p < 0.001). B. Relationship between macrolide use in the outpatient setting (horizontal axis) and prevalence of macrolide-resistant *S. pyogenes* (vertical axis) in 14 industrialized countries. A regression line was fitted with 95% confidence bands (r = 0.71; p = 0.004).

Total use of outpatient antibiotics varied from 9.0 DDD/1,000 inhabitants per day in the Netherlands to 36.5 DDD/1,000 inhabitants/day in France (Figure 1). Macrolide use varied from 1.0 DDD/1,000 inhabitants/day in Sweden to 6.0 DDD/1,000 inhabitants/day in France (Figure 2A). Average prevalence of PNSP also showed marked differences, as shown in Figure 1. It was lowest in Scandinavian countries (1% in Norway, 2% in Denmark, 4% in Sweden) and the Netherlands (1%); of medium level in Germany (7%), the United Kingdom (11%), Austria (12%), Belgium, and Italy (both 13%); and high in Portugal (29%), Greece (31%), United States (34%), France (43%), and Spain (50%). Figure 2A illustrates the prevalence of MRSP, which was low in Scandinavia and the Netherlands (3%–5%), medium level in Germany and Portugal (both 9%), and high in Belgium (43%), Spain (36%), and France (53%). MRGAS were distributed in a similar pattern (Figure 2B), with northern European countries showing low levels (0%–4%) and Greece, Italy, and Spain demonstrating the highest levels (29%–38%). Total antibiotic use and prevalence of PNSP were significantly correlated (r = 0.75; p < 0.001; Figure 1), as were macrolide use and prevalence of MRSP (r = 0.88; p < 0.001; Figure 2A) and macrolide use and prevalence of MRGAS (r = 0.71; p = 0.004; Figure 2B).

## Conclusions

This report correlates national outpatient data about antibiotic use with prevalence of antibiotic-resistant *S. pneumoniae* and *S. pyogenes* in Europe, North America, and Australia. The ecologic study design chosen allowed us to evaluate the effect of antibiotic consumption on resistance rates on a national level. A strong relationship was documented between total volume of antibiotic consumption and prevalence of PNSP. An almost linear association existed between macrolide use and proportion of MRSP, a biologically plausible finding that has previously been documented on a smaller scale (*6*). A weaker, but still significant correlation was found for MRGAS.

We found large differences in resistance rates, even in neighboring countries. A variety of factors are responsible for this, but the selective pressure exerted by inappropriately used antibiotics is likely the most important (*35*). Indeed, volume of antibiotic use varies widely between countries (*12*). In previous cross-country comparisons of Germany, France, and the United States (*5*,*30*), we suggested that socioeconomic, cultural, and behavioral determinants have a major impact on outpatient antibiotic prescribing practices and resistance prevalence in respiratory pathogens on a national level. In addition, healthcare policies play an important role. In Denmark, for instance, an excessive use of tetracycline was noted, mainly for respiratory tract infections. This situation was corrected by removing subsidization for tetracycline, which resulted in a decrease in its use; this decrease was followed by a parallel decrease in tetracycline resistance among *S. pneumoniae* and other streptococci (*36*).

Antibiotic use exerts selective pressure on resistance in respiratory pathogens in several ways (*35*). Any type of recent antibiotic treatment (not only β-lactam agents) can select PNSP by inhibiting susceptible commensal flora and eradicating penicillin-susceptible pneumococci, thereby indirectly promoting the transmission of PNSP and increasing the prevalence of PNSP in a community or country (*9*). However, whether decreasing antibiotic use in the community will have a sustained impact on resistance rates is unclear. Antibiotic resistance may take longer to return to previous levels than the time it took for antibiotic resistance to increase after excessive antibiotic use. In Finland in the 1990s antibiotic resistance decreased slowly with substantially reduced antibiotic consumption after an initial rapid increase; such situations may lead to different correlations between use and resistance, depending on whether resistance is rising or falling (*32*).

This ecologic study has some limitations. First, antibiotic sales data cannot be used synonymously with antibiotic exposure without highlighting the problem of patient compliance, which is very difficult to quantify. However, in case of low compliance, antibiotics may be used later, and this self-medication may also contribute to resistance. Second, large variations in resistance are typically found, depending on the examined age group, with highest resistance rates and largest amounts of antibiotic consumption usually in children. Due to the study design chosen, we could not account for these differences. Third, no single surveillance study was identified that provided sufficient data on national resistance rates for all countries. Therefore, considerable heterogeneity was found among the quality and characteristics of the included surveillance studies. We tried to minimize this bias by excluding publications not meeting certain standards or examining only selected patient populations. Fourth, the type and number of tested organisms varied widely. Thus, sampling bias may have influenced the results of this study. Finally, data are lacking regarding the maximum time lag between antibiotic consumption in the community and possible changes in resistance patterns on a national level. A period of 1 to 2 years seems plausible, although more rapid changes can be observed.

Further studies should be undertaken to extend our analyses to Asian countries that are demonstrating high rates of streptococcal resistance (*31*). Moreover, monitoring of antimicrobial resistance should be continued on both the regional and national level. An important role may be played by networks such as the European Antimicrobial Resistance Surveillance System. At the same time, disclosure of sales data through pharmaceutical records may help facilitate research in this area. In summary, the data presented in this ecologic analysis suggest an important association between antibiotic consumption and resistance in streptococcal infections and lend support to the validity of efforts by professional organizations and policymakers to discourage overuse of antibiotics in the community.

## References

[1] Livermore DM. Bacterial resistance: origins, epidemiology, and impact. Clin Infect Dis. 2003;36(Suppl 1):S11–23. 10.1086/344654 12516026

[2] McGowan JE Jr. Antimicrobial resistance in hospital organisms and its relation to antibiotic use. Rev Infect Dis. 1983;5:1033–48. 6318289 10.1093/clinids/5.6.1033

[3] Harbarth S, Harris AD, Carmeli Y, Samore MH. Parallel analysis of individual and aggregated data on antibiotic exposure and resistance in gram-negative bacilli. Clin Infect Dis. 2001;33:1462–8. 10.1086/322677 11588690

[4] Seppälä H, Klaukka T, Vuopio-Varkila J, Muotiala A, Helenius H, Lager K, The effect of changes in the consumption of macrolide antibiotics on erythromycin resistance in group A streptococci in Finland. N Engl J Med. 1997;337:441–6. 10.1056/NEJM199708143370701 9250845

[5] Harbarth S, Albrich W, Goldmann DA, Huebner J. Control of multiply resistant cocci: do international comparisons help? Lancet Infect Dis. 2001;1:251–61. 10.1016/S1473-3099(01)00120-7 11871512

[6] Pihlajamaki M, Kotilainen P, Kaurila T, Klaukka T, Palva E, Huovinen P. Macrolide-resistant *Streptococcus pneumoniae* and use of antimicrobial agents. Clin Infect Dis. 2001;33:483–8. 10.1086/322735 11462184

[7] Bronzwaer SL, Cars O, Buchholz U, Molstad S, Goettsch W, Veldhuijzen IK, A European study on the relationship between antimicrobial use and antimicrobial resistance. Emerg Infect Dis. 2002;8:278–82. 10.3201/eid0803.010192 11927025 PMC2732471

[8] McCormick AW, Whitney CG, Farley MM, Lynfield R, Harrison LH, Bennett NM, Geographic diversity and temporal trends of antimicrobial resistance in *Streptococcus pneumoniae* in the United States. Nat Med. 2003;9:424–30. 10.1038/nm839 12627227

[9] Lipsitch M. Measuring and interpreting associations between antibiotic use and penicillin resistance in *Streptococcus pneumoniae.* Clin Infect Dis. 2001;32:1044–54. 10.1086/319604 11264033

[10] McManus P, Hammond ML, Whicker SD, Primrose JG, Mant A, Fairall SR. Antibiotic use in the Australian community, 1990–1995. Med J Aust. 1997;167:124–7. 9269265 10.5694/j.1326-5377.1997.tb138809.x

[11] Bergan T. Antibiotic usage in Nordic countries. Int J Antimicrob Agents. 2001;18:279–82. 10.1016/S0924-8579(01)00382-X 11673043

[12] Cars O, Mölstad S, Melander A. Variation in antibiotic use in the European Union. Lancet. 2001;357:1851–3. 10.1016/S0140-6736(00)04972-2 11410197

[13] Cižman M, Pokorn M, Paragi M. Antimicrobial resistance of invasive *Streptococcus pneumoniae* in Slovenia from 1997 to 2000. J Antimicrob Chemother. 2002;49:582–4. 10.1093/jac/49.3.582 11864967

[14] Natsch S, Hekster YA, de Jong R, Heerdink ER, Herings RM, van der Meer JW. Application of the ATC/DDD methodology to monitor antibiotic drug use. Eur J Clin Microbiol Infect Dis. 1998;17:20–4. 10.1007/BF01584358 9512177

[15] Chen DK, McGeer A, de Azavedo JC, Low DE. Decreased susceptibility of *Streptococcus pneumoniae* to fluoroquinolones in Canada. N Engl J Med. 1999;341:233–9. 10.1056/NEJM199907223410403 10413735

[16] Ortqvist A. Pneumococcal disease in Sweden: experiences and current situation. Am J Med. 1999;107:44S–9S. 10.1016/S0002-9343(99)00101-1 10451008

[17] Bandak SI, Turnak MR, Allen BS, Bolzon LD, Preston DA. Assessment of the susceptibility of *Streptococcus pneumoniae* to cefaclor and loracarbef in 13 countries. J Chemother. 2000;12:299–305. 10949979 10.1179/joc.2000.12.4.299

[18] Descheemaeker P, Chapelle S, Lammens C, Hauchecorne M, Wijdooghe M, Vandamme P, Macrolide resistance and erythromycin resistance determinants among Belgian *Streptococcus pyogenes* and *Streptococcus pneumoniae* isolates. J Antimicrob Chemother. 2000;45:167–73. 10.1093/jac/45.2.167 10660498

[19] Felmingham D, Gruneberg RN. The Alexander Project 1996-1997: latest susceptibility data from this international study of bacterial pathogens from community-acquired lower respiratory tract infections. J Antimicrob Chemother. 2000;45:191–203. 10.1093/jac/45.2.191 10660501

[20] Granizo JJ, Aguilar L, Casal J, Dal-Re R, Baquero F. *Streptococcus pyogenes* resistance to erythromycin in relation to macrolide consumption in Spain (1986–1997). J Antimicrob Chemother. 2000;46:959–64. 10.1093/jac/46.6.959 11102415

[21] Jones RN, Pfaller MA. Macrolide and fluoroquinolone (levofloxacin) resistances among *Streptococcus pneumoniae* strains: significant trends from the SENTRY Antimicrobial Surveillance Program (North America, 1997–1999). J Clin Microbiol. 2000;38:4298–9. 11185065 10.1128/jcm.38.11.4298-4299.2000 PMC87592

[22] Marchese A, Schito GC. Resistance patterns of lower respiratory tract pathogens in Europe. Int J Antimicrob Agents. 2000;16:S25–9. 10.1016/S0924-8579(00)00302-2 11137405

[23] Sahm DF, Jones ME, Hickey ML, Diakun DR, Mani SV, Thornsberry C. Resistance surveillance of *Streptococcus pneumoniae, Haemophilus influenzae* and *Moraxella catarrhalis* isolated in Asia and Europe, 1997-1998. J Antimicrob Chemother. 2000;45:457–66. 10.1093/jac/45.4.457 10747822

[24] Schito GC, Debbia EA, Marchese A. The evolving threat of antibiotic resistance in Europe: new data from the Alexander Project. J Antimicrob Chemother 2000;46 Suppl T1:3–9. 10.1093/oxfordjournals.jac.a020891 10997593

[25] Cižman M, Pokorn M, Seme K, Orazem A, Paragi M. The relationship between trends in macrolide use and resistance to macrolides of common respiratory pathogens. J Antimicrob Chemother. 2001;47:475–7. 10.1093/jac/47.4.475 11266425

[26] Kristiansen BE, Sandnes RA, Mortensen L, Tveten Y, Vorland L. The prevalence of antibiotic resistance in bacterial respiratory pathogens from Norway is low. Clin Microbiol Infect. 2001;7:682–7. 11843910 10.1016/s1198-743x(14)64110-0

[27] de Neeling AJ, Overbeek BP, Horrevorts AM, Ligtvoet EE, Goettsch WG. Antibiotic use and resistance of *Streptococcus pneumoniae* in The Netherlands during the period 1994–1999. J Antimicrob Chemother. 2001;48:441–4. 10.1093/jac/48.3.441 11533014

[28] Perez-Trallero E, Fernandez-Mazarrasa C, Garcia-Rey C, Bouza E, Aguilar L, Garcia-de-Lomas J, Antimicrobial susceptibilities of 1,684 *Streptococcus pneumoniae* and 2,039 *Streptococcus pyogenes* isolates and their ecological relationships: results of a 1-year (1998–1999) multicenter surveillance study in Spain. Antimicrob Agents Chemother. 2001;45:3334–40. 10.1128/AAC.45.12.3334-3340.2001 11709305 PMC90834

[29] Canton R, Loza E, Morosini MI, Baquero F. Antimicrobial resistance amongst isolates of *Streptococcus pyogenes* and *Staphylococcus aureus* in the PROTEKT antimicrobial surveillance programme during 1999-2000. J Antimicrob Chemother 2002;50 Suppl S1:9–24. 10.1093/jac/dkf811 12239225

[30] Harbarth S, Albrich W, Brun-Buisson C. Outpatient antibiotic use and prevalence of antibiotic-resistant pneumococci in France and Germany: a sociocultural perspective. Emerg Infect Dis. 2002;8:1460–7. 12498664 10.3201/eid0812.010533 PMC2738507

[31] Felmingham D, Reinert RR, Hirakata Y, Rodloff A. Increasing prevalence of antimicrobial resistance among isolates of *Streptococcus pneumoniae* from the PROTEKT surveillance study, and comparative in vitro activity of the ketolide, telithromycin. J Antimicrob Chemother 2002; (Suppl S1):25–37. 10.1093/jac/dkf808 12239226

[32] Huovinen P. Macrolide-resistant group A streptococcus--now in the United States. N Engl J Med. 2002;346:1243–5. 10.1056/NEJM200204183461613 11961156

[33] Konradsen HB, Kaltoft MS. Invasive pneumococcal infections in Denmark from 1995 to 1999: epidemiology, serotypes, and resistance. Clin Diagn Lab Immunol. 2002;9:358–65. 11874878 10.1128/CDLI.9.2.358-365.2002 PMC119932

[34] Sa-Leao R, Vilhelmsson SE, de Lencastre H, Kristinsson KG, Tomasz A. Diversity of penicillin-nonsusceptible *Streptococcus pneumoniae* circulating in Iceland after the introduction of penicillin-resistant clone Spain(6B)-2. J Infect Dis. 2002;186:966–75. 10.1086/343769 12232837

[35] Lipsitch M, Samore MH. Antimicrobial use and antimicrobial resistance: a population perspective. Emerg Infect Dis. 2002;8:347–54. 10.3201/eid0804.010312 11971765 PMC2730242

[36] Monnet DL. Consommation d’antibiotiques et resistance bacterienne. Ann Fr Anesth Reanim. 2000;19:409–17. 10874442 10.1016/s0750-7658(00)90211-9

